# Evaluation of Genetic Variations in Organic Cationic Transporter 3 in Depressed and Nondepressed Subjects

**DOI:** 10.5402/2011/161740

**Published:** 2011-08-14

**Authors:** Nina Hengen, Mitsi H. Lizer, Robert S. Kidd

**Affiliations:** Bernard J. Dunn School of Pharmacy, Shenandoah University, 1775 North Sector Court, Winchester, VA 22601, USA

## Abstract

Organic cationic transporter 3 (*OCT3*, SLS22A3) has only recently emerged as one of the regulators of monoaminergic neurotransmission, which plays a critical role in the pathogenesis of depression and is a potential new antidepressant drug target. *OCT3* single-nucleotide polymorphisms (SNPs) have been investigated for their association with psychiatric disorders such as methamphetamine use disorder and obsessive-compulsive disorder in children and adolescents, but not depression. This study was designed to evaluate the allele frequencies of seven *OCT3* SNPs in a US Caucasian depressed population and compare these frequencies with a control group of nondepressed subjects. Informed consent and a DNA sample were obtained from 157 subjects and analysis was performed using real-time PCR. Allele and genotype frequencies were compared using a *t*-test and the Pearson chi-square analysis, respectively. There were no significant differences in *OCT3* allele or genotype frequencies between the depressed and non-depressed groups for all seven SNPs evaluated.

## 1. Introduction

Depression affects up to 20% of the world population and is one of the top ten causes of morbidity and mortality worldwide [1]. Even though the precise neurobiological processes causing depression are not yet completely understood, it is certain that monoamine neurotransmission plays an important role [2], as the vast majority of currently available antidepressants alter serotonergic, noradrenergic, and/or dopaminergic neurotransmission [3].

Monoamine neurotransmission is regulated by the synaptic levels of neurotransmitters serotonin (5-HT), norepinephrine (NE), and dopamine (DA). These levels are, in turn, determined by the activity of monoamine transporters [4]. Two uptake mechanisms are responsible for removal of synaptic monoamines. The high-affinity, low-capacity, substrate-specific, and Na^+^- and Cl^−^-dependent system consists of serotonin (SERT), norepinephrine (NET), and dopamine (DAT) transporters [4–6]. On the other hand, a low-affinity, high-capacity, multispecific, and Na^+^- and Cl^−^-independent system includes organic cation transporters (*OCT*) 1, 2, and 3 (also known as solute carrier family 22 members 1–3, (*SLC22A*)1–3) [7]. All three *OCTs* are capable of transporting multiple endogenous and exogenous organic cations, including neurotransmitters 5-HT, NE, epinephrine, DA, and histamine [7]. 

*OCTs* show a broad tissue distribution, with expression in the CNS and peripheral tissues in both humans and rodents [7]. While all three OCT subtypes are expressed in the brain [7], *OCT3* is preferentially expressed in brain regions that belong to monoamine pathways, such as raphe nuclei, striatum, ventral tegmental area, locus coeruleus, hippocampus, and cortex [8–10], as well as the dorsomedial hypothalamus which is the brain region involved in stress regulation of monoamine neurotransmission [11, 12]. Considering the important role of stress in the pathogenesis of depression [13], it is interesting that *OCT3* is more sensitive to inhibition by the stress hormone corticosterone than the other two *OCTs* [12, 14–16].

 The fact that *OCT3* transports 5-HT, NE, and DA [7] and is associated with monoamine pathways [8–10], points towards *OCT3* playing a role in the regulation of monoamine neurotransmission. Indeed, *OCT3*-deficient mice show altered monoamine neurotransmission [9, 17], anxiety-related behavior [9, 18], stress response [9], and response to psychostimulants [9]. Therefore, *OCT3* could be a candidate gene for association with neuropsychiatric disorders, such as depression, where monoamine neurotransmission plays a critical role.

 Studies investigating the association of different monoamine transporter gene polymorphisms with depression have primarily focused on SERT and NET gene polymorphisms [19–23]. However, the association between *OCT3* gene polymorphisms and depression has not been examined so far. Recently, *OCT3* gene single-nucleotide polymorphisms (SNPs) have been investigated for their association with several psychiatric and nonpsychiatric disorders, such as coronary artery disease [24], diabetic nephropathy, and hypertension [25]. Psychiatric disorders examined so far are methamphetamine use disorder [26] and obsessive-compulsive disorder in children and adolescents [27], but not depression. Therefore, the goal of this study was to evaluate seven *OCT3* gene SNPs in a US Caucasian depressed population and compare these allele frequencies and resulting genotypes with a control group of nondepressed subjects.

## 2. Methods

### 2.1. Subjects

This cross-sectional study utilized subjects currently being treated for depression at a community-based psychiatric practice and an ambulatory care clinic (depressed group). The nondepressed control group was recruited from the same ambulatory care clinic and the community. Subjects in the depressed group were identified by either chart review or referred by their physician with inclusion criteria of a documented diagnosis of depression and no other Axis I or Axis II diagnosis. All self-reported as Caucasian. Patients under the age of 18 years old or with a concurrent diagnosis of bipolar disorder were excluded. Subjects in the nondepressed (control) group were self-referred and reported no previous nonpharmacologic or pharmacologic treatment of depression or bipolar disorder. All self-reported as Caucasian. Axis II diagnosis in the control group was unknown. Subjects under the age of 18 years old were also excluded from the control group. No other medical comorbidity information was collected for either group. All subjects signed an informed consent, and two cheek swab samples were obtained for DNA collection. This study was approved by the Shenandoah University Institutional Review Board prior to its commencement.

### 2.2. Genotyping

Genomic DNA (gDNA) was isolated from the cheek swab samples using a Qiagen QiaCube automated DNA isolation instrument (Qiagen Inc, Chatsworth, Calif, USA) following the manufacturer's protocol and then frozen at −20°C until the time of genotyping. A total of seven *OCT3* gene SNPs were selected for this study: SNP1 (rs1810126), SNP2 (rs204832), SNP3 (rs2048327), SNP4 (rs665185), SNP5 (rs4709426), SNP6 (rs3106164), and SNP7 (rs 2292334). SNPs 3, 4, 5, 6, and 7 have been previously assessed for their correlation with methamphetamine use disorder in a Japanese population [26]. SNPs 1 and 7 have been found to be present in Caucasian population [28]. In addition, SNPs 1, 2, 3, and 7 have been found to be associated with reduced *OCT3* mRNA levels in the human liver [29]. The genotyping was performed on an Applied Biosystems 7300 real-time PCR (Applied Biosystems, Foster City, Calif, USA) with commercially available, validated TaqMan SNP Genotyping Assays for *OCT3* (Applied Biosystems, Foster City, CA). The genotyping assays used included nonlabeled primers and one wild-type and one variant allele-specific fluorescent TaqMan labeled oligonucleotide probes. PCR was performed with a reaction volume of 10 *μ*L, which included 4.75 *μ*L of TaqMan Genotyping Master Mix (Applied Biosystems), 0.5 *μ*L of 20x SNP Genotyping Assay, 3.75 *μ*L of DEPC H_2_O, and 1.0 *μ*L of gDNA. The PCR cycling conditions were as follows: 1 cycle of 50°C for 2 minutes, followed by 1 cycle of 95°C for 10 minutes, and 50 cycles of 92°C for 15 seconds and 60°C for 90 seconds. Allelic discrimination was evaluated by measuring endpoint fluorescence intensity. These results were analyzed with SDS software Version 1.3 (Applied Biosystems), and the corresponding genotypes were assigned.

### 2.3. Statistical Analysis

A power calculation was performed, and it was estimated that 72 subjects would be required in each group to detect a clinically significant difference of 20% in each allele frequency, with a power of 80% and a significance level of 5%. Deviation from the Hardy-Weinberg equilibrium was tested by allele counting and evaluated by the chi-square (*χ* ^2^) test. Allele frequencies were first evaluated with Levene's test for equality of variances and then compared between the two groups using the Student *t*-test. Genotypes were compared between groups using the Pearson *χ* ^2^ test. A *P* < 0.05 was considered statistically significant. All statistical analyses were performed using PASW v18.0 (SPSS Inc. Chicago, Il, USA).

## 3. Results

A total of 157 subjects were recruited into the study. There were 82 subjects in the depressed group (28.6% males and 71.4% females) and 75 subjects in the control group (37.8% males and 62.2% females). The *OCT3* genotype frequencies for all seven SNPs examined did not deviate from the Hardy-Weinberg equilibrium in either the depressed or nondepressed control group. Genotype and allele frequencies for those seven SNPs in depressed patients and nondepressed control subjects are summarized in Table 1. No significant difference was found in either genotype prevalence or allele frequencies between the depressed and nondepressed control groups (Table 1). In addition, this study found none of the SNPs evaluated to be in complete linkage disequilibrium with one another.

## 4. Discussion

To the authors' knowledge, this is the first study to examine the potential association of *OCT3* gene polymorphisms with depression. We evaluated the allele frequencies of seven *OCT3* gene SNPs in the US Caucasian patients actively being treated for depression and compared these frequencies with a control group of nondepressed subjects. No significant difference was found in allele frequencies between the depressed and nondepressed groups. 

SNPs 3, 5, and 6 have been found to be associated with methamphetamine dependence disorder in a Japanese population [26]. That study found the major (M) allele frequency in the control group to be 50.9% for SNP 3, 59.8% for SNP 5, and 69.1% for SNP 6. In our study, M allele frequencies in the control group were 59.8% for SNP 3, 52.4% for SNP 5, and 57.3% for SNP 6. Lazar et al. [28] examined *OCT3* gene polymorphism in 100 Caucasian individuals and found the frequency of the M allele to be 64% for SNP1 and 63.5% for SNP 7. Our findings for M allele frequency in the control group are 54.7% for SNP 1 and 55.3% for SNP 7. Therefore, the M allele frequencies we found in the US Caucasian population are relatively consistent with frequencies found in different geographic and ethnic groups. 

SNPs 1, 2, 3, and 7 have been found to be associated with reduced *OCT3* mRNA levels in the human liver [29]. However, at this point it is not known whether this translates to reduced *OCT3* mRNA levels or altered *OCT3* function in the brain for these particular SNPs. A recent study by Sakata et al. [30] analyzed the functional properties of five *OCT3* SNPs resulting in the amino acid changes using a transient expression system. The study found decreased *OCT3* uptake capacity for three of those five SNPs tested. However, the minor allele frequency for these three SNPs is extremely low (less than 1%), which leads us not to pursue evaluation of these three SNPs at this time. Obviously, further studies using these and additional SNPs are needed.

Several limitations of the present study should be noted. First, with the sample size of 82 subjects in the depressed group and 75 subjects in the control group, it is possible to detect a difference of 20% in each allele frequency, but not differences lower than 20%. Second, our study did not include the data on the severity of depression in the depressed group or the information on existence of other medical comorbidities in either group.

Recently, *OCT3* has received considerable attention in regard to its role in the regulation of monoamine neurotransmission under both basal conditions [31] and during stress [32, 33]. In addition, studies using animal models of depression demonstrated antidepressant effects of both pharmacological [34] and antisense blockade [35] of *OCT3*. As treatment-resistant depression continues to present a significant clinical problem [36], and there is an ongoing search for new antidepressant drugs, these data support the notion of *OCT3* as a potential new antidepressant therapy target [31, 37].

In conclusion, this study is, to our knowledge, the first to compare the allele and genotype frequencies of seven *OCT3* gene SNPs between a US Caucasian depressed population and a control group of nondepressed subjects. No significant difference was found between these two groups for any of the SNPs examined. However, further studies are needed to verify these findings.

## Figures and Tables

**Table 1:** Genotype and allele distributions of *OCT3* SNPs in depressed and nondepressed subjects.

SNP	Sample	Genotype				Allele frequency		
		M/M	M/m	m/m	*P* value	M	m	*P* value
SNP1	Depressed	30	38	14	0.433	0.598	0.442	0.364
rs1810126	Control	26	30	19		0.547	0.453	

SNP2	Depressed	29	39	14	0.442	0.591	0.409	0.425
rs2048327	Control	26	30	19		0.547	0.453	

SNP3	Depressed	29	40	13	0.163	0.598	0.402	0.207
rs3088442	Control	25	29	21		0.527	0.473	

SNP4	Depressed	21	44	17	0.737	0.524	0.476	0.694
rs665185	Control	23	36	16		0.547	0.453	

SNP5	Depressed	22	42	18	0.954	0.524	0.476	0.783
rs4709426	Control	21	39	15		0.54	0.46	

SNP6	Depressed	29	37	16	0.791	0.579	0.421	0.916
rs3106164	Control	28	30	17		0.573	0.427	

SNP7	Depressed	30	38	14	0.403	0.598	0.402	0.430
rs2292334	Control	27	29	19		0.553	0.447	

M: major allele; m: minor allele.
